# Reduced mean platelet volume levels predict shorter survival in patients with resectable pancreatic ductal adenocarcinoma and type 2 diabetes

**DOI:** 10.1186/s12876-020-01225-y

**Published:** 2020-05-11

**Authors:** Ji-bin Yin, Na Li, Ming-ming Cui, Xin Wang, Rui-tao Wang

**Affiliations:** 1 Department of Gastroenterology, The Second Affiliated Hospital of Harbin Medical University, Harbin Medical University, No. 246 Xuefu ST, Nangang District, Harbin, 150086 Heilongjiang China; 2 Department of Internal Medicine, Harbin Medical University Cancer Hospital, Harbin Medical University, NO.150 Haping ST, Nangang District, Harbin, 150081 Heilongjiang China

**Keywords:** Pancreatic ductal adenocarcinoma, Type 2 diabetes mellitus, Survival, Mean platelet volume, Prognosis

## Abstract

**Background:**

Type 2 diabetes mellitus (T2DM) increased the risk of developing pancreatic cancer. Pancreatic ductal adenocarcinoma (PDAC) is the most common neoplastic disease originating from the pancreas. Increasing evidence indicates that platelets activation plays a prominent role in tumor and T2DM. Mean platelet volume (MPV) is an indicator of activated platelets and is altered in several cancers. The current study aimed to evaluate the prognostic role of MPV in resectable PDAC patients with T2DM.

**Methods:**

Eight hundred and three patients with PDAC were included in this retrospective study. We determined the optimal cutoff value of MPV for 5-year overall survival (OS) using the receiver operating characteristic (ROC) method. The associations between MPV levels and clinical characteristics were analyzed. Kaplan-Meier survival analysis and Cox’s proportional hazard regression model were used to evaluate the prognostic value of MPV for OS.

**Results:**

Compared to the PDAC patients without T2DM, MPV levels were significantly higher in the PDAC patients with T2DM. Moreover, MPV was significantly associated with the differentiation between T2DM and non-T2DM. In addition, Kaplan-Meier analysis found that patients with low MPV levels had a poorer 5-year OS than patients with high MPV levels in diabetic patients. Multivariate analyses revealed that MPV was an independent prognostic factor for OS in patients with T2DM. However, the independent prognostic role of MPV was not observed in patients without T2DM.

**Conclusion:**

MPV independently predicts poor survival in PDAC patients with T2DM. Prospective studies are required to confirm the role of MPV in PDAC.

## Background

Pancreatic ductal adenocarcinoma (PDAC) is the most common type of cancer originating from the pancreas and the fourth leading cause of cancer-related deaths [1]. PDAC has a 5-year overall survival (OS) of 8% for all stages combined [2]. The existence of type 2 diabetes mellitus (T2DM) reflects pancreatic dysfunction and facilitates pancreatic tumorigenesis [3]. In addition, T2DM predicts worse survival outcomes in patients undergoing pancreatic resection [4]. Although the progress has been made in therapeutic approaches, however, the survival rate of PADC patients remains low. Therefore, effective molecular targets better predicting survival outcome are urgently needed in PDAC.

Platelets are implicated in tumor biology through the direct interaction with tumor cells [5]. Thrombocytosis correlates with worse overall survival in gastric cancer, pancreatic cancer, colorectal cancer, endometrial cancer, and ovarian cancer [6–10]. However, platelet number is related to production and consumption. Platelet count is normal when pro-inflammatory state and efficient compensatory mechanisms coexisted [11].

Mean platelet volume (MPV) reflects platelet size and indicates platelet activation [12]. Platelet activation acted as an active role in cancer progression and metastasis [13]. Altered MPV levels were observed in several malignancies, such as gastric cancer, ovarian cancer, lung cancer, colorectal cancer, and breast cancer [14–18]. Moreover, MPV is associated with a number of diabetic complications such as diabetic macular edema, microalbuminuria, coronary severity scores, and stroke [19–23]. However, to our knowledge, there has been no report describing its value in PDAC patients with T2DM.

The study aimed to examine the predictive significance of MPV in PDAC patients with T2DM.

### Methods

#### Study population

This study consisted of 803 consecutive PDAC patients (median age 60.0 years, range 21–78 years) in Harbin Medical University Cancer Hospital from January 2010 to December 2013. All patients underwent surgical resection. Patients with the following characteristics were included: 1) age > 21 years; 2) pathologically confirmed PDAC; 3) no distant metastasis; 4) no chemotherapy or radiotherapy before operation. The exclusion criteria were as follows: 1) other malignancies diagnosed within 5 years; 2) acute infection; 3) hematological disorders, 4) treatment with acetylic salicylic acid; and 5) no complete follow-up data. The clinical staging was determined by TNM staging system of the American Joint Commission on Cancer (AJCC) 8th edition. Diabetes mellitus (DM) was defined as a prior diagnosis or fasting serum glucose was ≥ 7.0 mmol/L or random serum glucose was ≥ 11.1 mmol/L or based on medication. The time from diagnosis to death or last follow-up was measured as overall survival (OS). Follow-up was completed on December 31, 2018.

The protocol was approved by the ethical committee review board of Harbin Medical University Cancer Hospital. All patients involved in the study gave written consent for this study.

#### Statistical analysis

We used SPSS Statistics version 25.0 (SPSS Inc., Chicago, IL, USA) to analyze data. Continuous variables were presented as means ± SD or medians and compared with the Student’s *t* test or Mann-Whitney *U* test. Categorical variables were presented as numbers and percentages and compared with the χ^2^ test. Associations between prognostic factors and OS were estimated by the Kaplan–Meier method and assessed by the log-rank test. Cox’s proportional hazard regression model was used to assess the independent predictors for OS. The variables with a *P*-value less than 0.10 in univariate analysis were included in the multivariate Cox analysis. The cut-off value of MPV was determined using receiver-operating characteristics (ROC) curve analysis. A *p* value of < 0.05 was considered statistically significant.

## Results

Among the 803 patients, 487 (60.6) were men and 316 (39.4) were women, and the median age was 60.0 years (range 21–89). 202 PDAC patients were categorized as T2DM (median age was 61.5 years), and 601 PDAC patients were categorized as non-T2DM (median age was 59.0 years). The baseline characteristics of the patients are listed in Table 1. Compared with the patients without T2DM, the patients with T2DM were older and had higher WBC and MPV levels. Regional lymph node metastasis were more common in T2DM group. However, tumor location, tumor size, tumor differentiation, and postoperative adjuvant chemotherapy were not significantly different between the groups.

**Table 1:** The clinicopathological features among PDAC patients in relation to T2DM status

Variables	With T2DM	Without T2DM	*P* value
N	202	601	
Age (years)	61.7 (8.7)	58.2 (10.9)	< 0.001
Sex (male, %)	120 (59.4)	367 (61.1)	0.676
BMI (kg/m^2^)	23.4 (3.2)	23.1 (3.3)	0.183
Smoker (n, %)	61 (30.2)	206 (34.3)	0.287
Drinking (n, %)	44 (21.8)	113 (18.8)	0.356
FPG (mmol/L)	8.45 (6.70–11.10)	5.40 (4.95–6.10)	< 0.001
Albumin (g/L)	40.4 (6.7)	40.1 (6.4)	0.599
WBC (×10^9^/L)	7.38 (3.19)	6.72 (2.77)	0.005
Hemoglobin (g/dl)	129.0 (15.2)	129.9 (17.7)	0.512
Platelet count (×10^9^/L)	216.6 (87.2)	223.6 (78.0)	0.278
MPV (fL)	9.9 (1.9)	10.3 (2.0)	0.011
CA19–9 (IU/mL)			0.577
≤ 37	38 (18.8)	124 (20.6)	
> 37	164 (81.2)	477 (79.4)	
Tumor location			0.240
Head	135 (66.8)	374 (62.2)	
Body, tail	67 (33.2)	227 (37.8)	
Tumor differentiation			0.092
Well/moderate	170 (84.2)	533 (88.7)	
Poor	32 (15.8)	68 (11.3)	
Tumor size (cm)			0.156
≤ 4	158 (78.2)	497 (82.7)	
> 4	44 (21.8)	104 (17.3)	
Regional lymph node metastasis			< 0.001
N0	124 (61.4)	464 (77.2)	
N1	69 (34.2)	111 (18.5)	
N2	9 (4.5)	26 (4.3)	
Postoperative adjuvant chemotherapy			0.334
Yes	103 (51.0)	330 (54.9)	
No	99 (49.0)	271 (45.1)	

*T2DM* type 2 diabetes mellitus; *PDAC* pancreatic ductal adenocarcinoma; *BMI* body mass index; *FPG* fasting plasma glucose; *WBC* white blood cells; *MPV* mean platelet volume

**Fig. 1:**
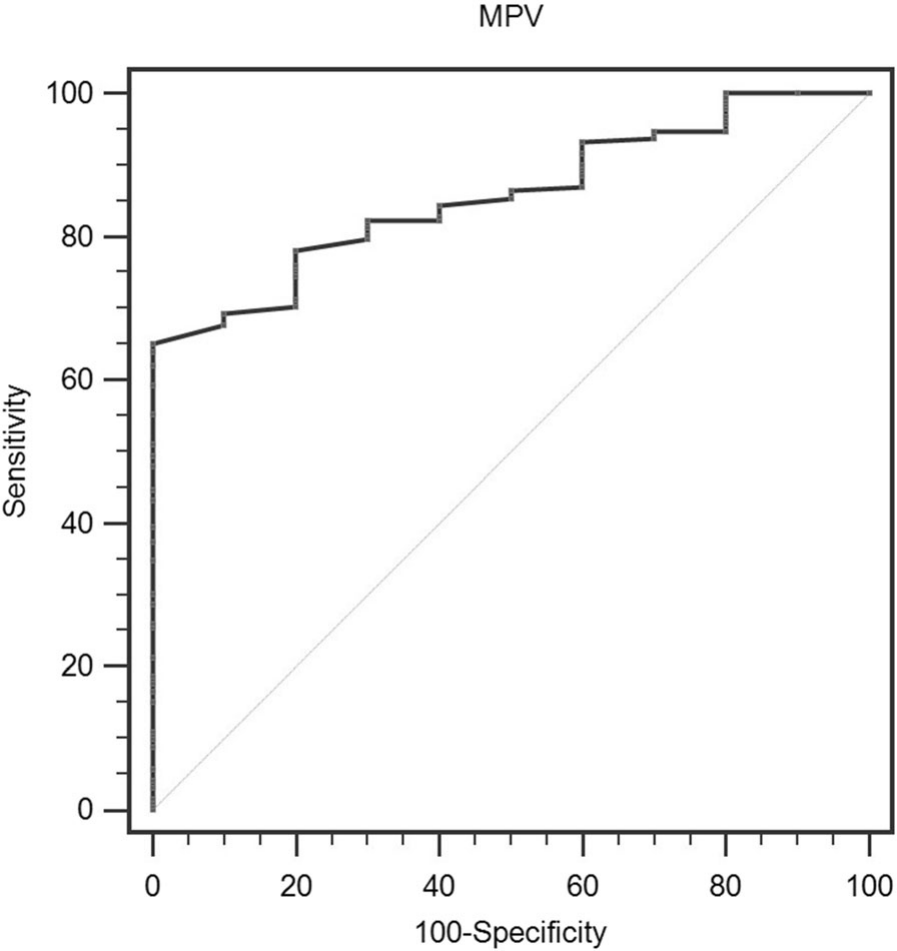
Optimized cut-off value for MPV using standard ROC curve analysis

The optimal cut-off value of MPV for OS prediction was 10.0 fL according to the ROC curve with 75.1% sensitivity and 71.4% specificity (Fig. 1). Patients were divided into two groups using this cut-off value. This cut-off value corresponds to an AUC of 0.857 (95% CI: 0.801–0.902, *p* <  0.001).

**Fig. 2:**
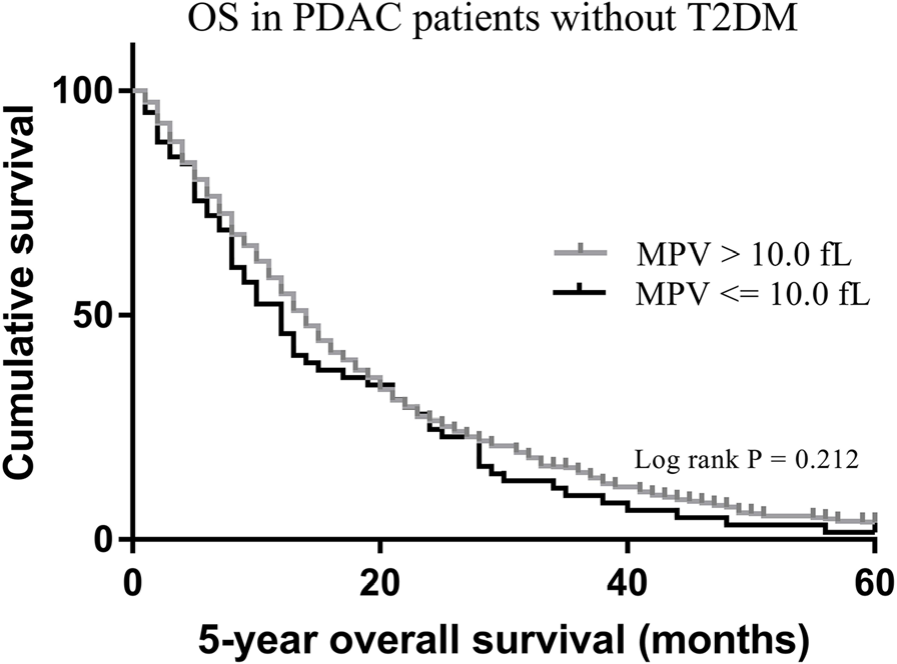
Kaplan-Meier analysis of overall survival in PDAC patients without T2DM

**Fig. 3:**
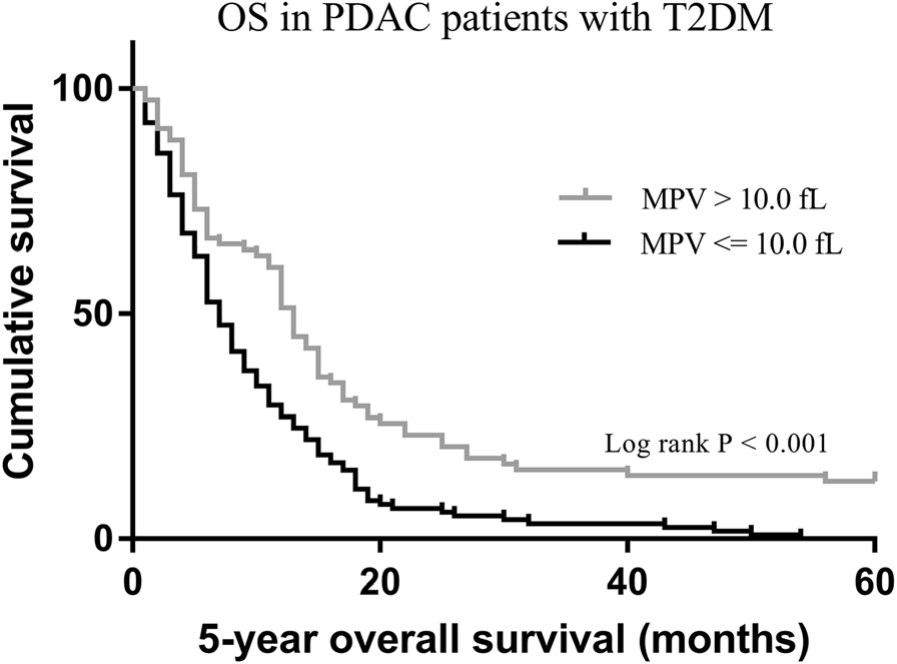
Kaplan-Meier analysis of overall survival in PDAC patients with T2DM

579 (96.3%) patients without T2DM had death events with a median follow up of 18.3 months. In this group without T2DM, the 5-year OS of patients according to different MPV levels did not show a significant difference (1.6% vs. 3.9%, *p* = 0.212) (Fig. 2). 192 (95.0%) patients with T2DM had death events with a median follow up of 13.0 months. In this group with T2DM, patients with low MPV levels (≤ 10.0 fL) showed significantly shorter OS compared to those with high MPV levels (> 10.0 fL) (0% vs. 12.7%, *p* <  0.001) (Fig. 3).

The risk factors for distinguishing T2DM from non-T2DM were evaluated using logistic regression analysis in Table 2. Age, MPV, WBC, and regional lymph node metastasis were significantly associated with differentiation in the regression analysis (for MPV, β = 0.904; *p* = 0.024).

**Table 2:** Logistic regression analysis on baseline variables associated with T2DM

Variables	β	OR (95% CI)	*P*-value
Age (years)	1.033	1.016–1.050	< 0.001
WBC (×10^9^/L)	1.073	1.017–1.132	0.010
MPV (fL)	0.904	0.829–0.987	0.024
Regional lymph node metastasis			
(N1 + N2 versus N0)	2.069	1.460–2.932	< 0.001

Abbreviations: see to Table 1

On univariate Cox regression analysis, platelet count, CA19–9, tumor location, tumor size, tumor differentiation, regional lymph node metastasis, and postoperative adjuvant chemotherapy were significantly correlated with OS in patients with non-T2DM (Table 3). Gender, WBC, MPV, CA19–9, tumor size, tumor differentiation, regional lymph node metastasis, and postoperative adjuvant chemotherapy were significantly correlated with OS in patients with T2DM (Table 4).

**Table 3:** Univariate and multivariate analysis of overall survival in PDAC patients without T2DM

Variables	Univariate analysis		Multivariate analysis	
	HR (95% CI)	*P*-value	HR (95% CI)	*P*-value
Age (years) (> 60 versus ≤60)	1.139 (0.967–1.343)	0.120		
Gender (Male versus Female)	1.022 (0.864–1.207)	0.802		
BMI (kg/m^2^)	0.985 (0.961–1.010)	0.239		
Smoking status (Yes versus No)	0.941 (0.792–1.118)	0.489		
Drinking status (Yes versus No)	1.001 (0.812–1.234)	0.993		
FPG (mmol/L) (log-value)	1.273 (0.750–2.160)	0.371		
Albumin (g/L)	1.002 (0.989–1.015)	0.767		
Hemoglobin (g/dl)	1.003 (0.998–1.007)	0.234		
WBC (×10^9^/L)	0.995 (0.996–1.026)	0.756		
Platelet count (× 10^9^/L)	0.999 (0.998–1.000)	0.071	0.999 (0.998–1.001)	0.131
MPV (fL) (≤ 10.0 versus > 10.0)	1.183 (0.907–1.542)	0.216		
CA19–9 (IU/mL)				
(≤ 37 versus > 37)	1.843 (1.488–2.284)	< 0.001	1.757 (1.408–2.193)	< 0.001
Tumor location				
(Head versus Body, tail)	1.208 (1.020–1.431)	0.028	1.095 (0.915–1.311)	0.322
Tumor differentiation				
(Poor versus Well/ moderate)	1.709 (1.324–2.206)	< 0.001	1.809 (1.400–2.337)	< 0.001
Tumor size (cm)				
(> 4 versus ≤4)	1.302 (1.051–1.613)	0.016	1.229 (0.992–1.523)	0.059
Regional lymph node metastasis				
(N1 + N2 versus N0)	1.285 (1.060–1.559)	0.011	1.239 (1.021–1.504)	0.030
Chemotherapy (Yes versus No)	0.800 (0.679–0.943)	0.008	0.774 (0.654–0.916)	0.003

Abbreviations: see to Table 1

**Table 4:** Univariate and multivariate analysis of overall survival in PDAC patients with T2DM

Variables	Univariate analysis		Multivariate analysis	
	HR (95% CI)	*P*-value	HR (95% CI)	*P*-value
Age (years) (> 60 versus ≤60)	1.099 (0.826–1.462)	0.519		
Gender (Male versus Female)	1.289 (0.963–1.725)	0.087	1.083 (0.795–1.476)	0.614
BMI (kg/m^2^)	0.973 (0.927–1.022)	0.278		
Smoking status (Yes versus No)	1.052 (0.770–1.436)	0.750		
Drinking status (Yes versus No)	1.076 (0.766–1.513)	0.672		
FPG (mmol/L) (log-value)	1.165 (0.792–1.713)	0.439		
Albumin (g/L)	1.013 (0.989–1.037)	0.283		
Hemoglobin (g/dl)	0.998 (0.989–1.008)	0.706		
WBC (×10^9^/L)	1.053 (1.002–1.106)	0.043	1.032 (0.979–1.087)	0.241
Platelet count (× 10^9^/L)	1.000 (0.999–1.002)	0.629		
MPV (fL) (≤ 10.0 versus > 10.0)	1.914 (1.414–2.592)	< 0.001	1.801 (1.305–2.485)	< 0.001
CA19–9 (IU/mL)				
(≤ 37 versus > 37)	1.205 (0.903–1.609)	0.205	1.856 (1.122–3.070)	0.016
Tumor location				
(Head versus Body, tail)	1.053 (0.779–1.424)	0.736		
Tumor differentiation				
(Poor versus Well/ moderate)	2.014 (1.473–2.753)	< 0.001	1.180 (0.673–2.070)	0.563
Tumor size (cm)				
(> 4 versus ≤4)	1.460 (1.091–1.955)	0.011	1.385 (0.832–2.303)	0.210
Regional lymph node metastasis				
(N1 + N2 versus N0)	1.481 (1.105–1.983)	0.009	1.268 (0.944–1.704)	0.115
Chemotherapy (Yes versus No)	0.166 (0.119–0.232)	< 0.001	0.183 (0.128–0.261)	< 0.001

Abbreviations: see to Table 1

In multivariate analysis, CA19–9, tumor differentiation, regional lymph node metastasis, and postoperative adjuvant chemotherapy were independently associated with OS in patients with non-T2DM (Table 3). MPV, CA19–9, and postoperative adjuvant chemotherapy were independently associated with OS in patients with T2DM (Table 4).

## Discussion

Our study revealed three important clinical findings. First, patients with T2DM had higher MPV levels than those in patients without T2DM. Second, MPV was significantly associated with differentiation between T2DM from non-T2DM. Third, MPV is an independent prognostic factor for OS in PDAC patients with T2DM.

The relationship between pancreatic cancer and diabetes is complex because causal relationship between the two is not clear. T2DM is associated with an increased risk for PDAC [24]. Hyperinsulinemia reduces the production of insulin-like growth factor (IGF) binding protein and results in the increase of bioavailable IGF-1 [25]. IGF-1 receptor binding activates PI3K/Akt and Raf/MAPK pathways, which further stimulate the growth of pancreatic cancer cells and inhibits apoptosis [26]. On the other hand, IGF-1 promotes tumor cell invasion and inhibits tumor suppressor phosphatase and tensin homolog (PTEN) [27]. In addition, advanced glycation end products (AGEs)-receptor interactions promote cell proliferation through the overexpression of platelet-derived growth factor-B [28]. These studies provided some biologic evidence for the roles played by activated platelets.

Our results are consistent with the studies above and indirectly confirmed the key role played by platelet activation. Moreover, these findings are also in accordance with the current idea that anti-platelet therapy is considered to be a part of adjuvant treatment of cancer [5]. A recent study confirmed that aspirin lessens the ability of platelets and inhibits PADC cell proliferation [29].

The underlying mechanisms of MPV involved in PDAC are unclear. Chronic inflammation plays a crucial role in the development and progression of PADC. MPV indicates platelets activation and low MPV levels reflect enhanced consumption of larger platelets in inflammatory states [12]. Previous studies confirmed that low MPV levels are linked with high-grade inflammatory diseases and reverse after anti-inflammatory treatment [12]. A recent study demonstrated that activated platelets released ADP and ATP and promoted pancreatic cancer cell survival via increasing cytidine deaminase expression [30]. In addition, tumor-infiltrating platelets predict a poor surgical outcome in PDAC patients [31].

Our results revealed the potential clinical significance of evaluating PADC prognosis using activated platelets. A report found that thrombocytosis and C-reactive protein influenced pancreatic cancer patient prognosis [32]. A meta-analysis showed that increased preoperative platelet to lymphocyte ratio had an association with reduced OS in PDAC [33].

Our study has certain limitations. First, our study was a retrospective and single-center design study with small sample size. In addition, the optimal cut-off value of MPV for OS prediction of 10.0 fL at a sensitivity and specificity of 75.1 and 71.4% respectively requires independent validation. Second, the intrinsic mechanisms of MPV in PDAC need further investigation. Third, diabetes and pre-diabetes are frequently undiagnosed in PDAC patients. When tested for diabetes, the percentage of PDAC patients with DM is around 50%. This increases if pre-diabetes is also considered. Therefore the incidence of diabetes reported in this cohort is likely to be underestimated.

## Conclusions

MPV independently predicts poor survival in PDAC patients with T2DM. Prospective studies are required to confirm the role of MPV in PDAC.

## Data Availability

The data in this study available from the corresponding author on reasonable request.

## Abbreviations

IGF: Insulin-like growth factor
MPV: Mean platelet volume
PDAC: Pancreatic ductal adenocarcinoma
T2DM: Type 2 diabetes mellitus
WBC: White blood cells
